# Associations between epigenetic aging and diabetes mellitus in a Swedish longitudinal study

**DOI:** 10.1007/s11357-024-01252-7

**Published:** 2024-06-27

**Authors:** Daniel Wikström Shemer, Shayan Mostafaei, Bowen Tang, Nancy L Pedersen, Ida K Karlsson, Tove Fall, Sara Hägg

**Affiliations:** 1 Department of Medical Epidemiology and Biostatistics, Karolinska Institutet, 171 77 Stockholm, Sweden; 2 Molecular Epidemiology, Department of Medical Sciences, and Science for Life Laboratory, Uppsala University, Uppsala, Sweden

**Keywords:** Diabetes mellitus, Aging, Epigenetic clocks, Longitudinal study

## Abstract

Diabetes mellitus type 2 (T2D) is associated with accelerated biological aging and the increased risk of onset of other age-related diseases. Epigenetic changes in DNA methylation levels have been found to serve as reliable biomarkers for biological aging. This study explores the relationship between various epigenetic biomarkers of aging and diabetes risk using longitudinal data. Data from the Swedish Adoption/Twin Study of Aging (SATSA) was collected from 1984 to 2014 and included 536 individuals with at least one epigenetic measurement. The following epigenetic biomarkers of aging were employed: DNAm PAI-1, DNAmTL, DunedinPACE, PCHorvath1, PCHorvath2, PCHannum, PCPhenoAge, and PCGrimAge. Firstly, longitudinal analysis of biomarker trajectories was done. Secondly, linear correlations between the biomarkers and time to diabetes were studied within individuals developing diabetes. Thirdly, Cox proportional hazards (PH) models were used to assess the associations between these biomarkers and time of diabetes diagnosis, with adjustments for chronological age, sex, education, smoking, blood glucose, and BMI. The longitudinal trajectories of the biomarkers revealed differences between individuals with and without diabetes. Smoothened average curves for DunedinPACE and DNAm PAI-1 were higher for individuals with diabetes around the age 60–70, compared to controls. Likewise, DunedinPACE and DNAm PAI-1 were higher closer to diabetes onset. However, no significant associations were found between the epigenetic biomarkers of aging and risk of diabetes in Cox PH models. Our findings suggest the potential value of developing epigenetic biomarkers specifically tailored to T2D, should we wish to model and explore the potential for predicting the disease.

**Supplementary Information:**

The online version contains supplementary material available at 10.1007/s11357-024-01252-7.

## Background

By the year 2045, diabetes is projected to impact approximately 693 million people globally [1]. Within the realm of diabetes, we recognize two main types: type 1 (T1D) and type 2 (T2D), and it was not until 1997 that the international classification of diseases (ICD) codes formally distinguished between these two types. Type 2 diabetes, currently the most prevalent form, accounts for 94% of new cases of diabetes among individuals aged 40 to 100 years [2]. Type 2 diabetes is a chronic metabolic disorder characterized by the body’s resistance to insulin, leading to high blood glucose levels [3]. If untreated, it can lead to complications such as cardiovascular disease, nephropathy, retinopathy, and neuropathy [4]. Age has been identified as a major risk factor for T2D [5] and individuals with advanced T2D exhibit organ system dysfunction like that observed in aging [6–8]. For instance, individuals with diabetes are more susceptible to developing comorbidities such as coronary artery disease, sarcopenia, frailty, and mild cognitive impairment. Considering these associations, diabetes can be viewed as a pro-aging state [9].

Epigenetics refers to chemical modifications to the DNA machinery, including the addition of a methyl group to a cytosine-guanine nucleotide pair (CpG site) on the DNA molecule. These epigenetic alterations impact DNA transcription across various cells, tissues, and organs throughout an individual’s life. Notably, changes in DNA methylation levels at specific genomic loci are closely linked to age and serve as valuable components in age prediction models [10].

Currently, several DNA methylation age predictors, often referred to as biomarkers of aging, are utilized to gauge an individual’s biological age [11]. These biomarkers exhibit varying degrees of correlation with each other [11, 12]. It has been hypothesized that biomarkers with low intercorrelation capture different aspects of aging [13].

Furthermore, some of these biomarkers demonstrate a low signal-to-noise ratio and exhibit limited test-retest reliability [14, 15]. Efforts have been made to enhance the reliability of certain biomarkers by calculating principal component (PC) versions [12]. This method not only improves test-retest reliability but also enhances the predictability of longitudinal trajectories.

Epigenetic age acceleration has been linked with higher mortality rates and a range of age-related conditions, including T2D [10]. Previous research has pinpointed differentially methylated regions in pancreatic islet cells among individuals with T2D, contrasting them with healthy controls [16]. Furthermore, epigenome-wide analyses have illuminated associations between DNA methylation sites and the incidence of T2D [17]. Notably, a recent study suggests that accelerated epigenetic aging may be linked to T2D up to a decade before its clinical manifestation [18].

However, most existing studies that investigate the relationship between DNA methylation and T2D have been cross-sectional in design. The primary objective of our study is to examine the link between biomarkers of epigenetic aging and the risk of diabetes, leveraging comprehensive longitudinal data.

## Methods

### Study design and participants

The research adhered to the guidelines outlined in the Strengthening the Reporting of Observational Studies in Epidemiology (STROBE) recommendations during both its execution and reporting. For this study, we utilized data from the Swedish Adoption/Twin Study of Aging (SATSA), which was collected between 1984 and 2014 [19]. The SATSA dataset is nested within the Swedish Twin Registry [20]. To gather SATSA data, twins aged 50 or above were invited to participate in ten in-person testing (IPT) occasions. These IPTs occurred at intervals of approximately 3 years for IPT 3–5 and then every 2 years from IPT 7 onward. A total of 859 individuals participated in at least one IPT. During these sessions, we collected blood samples for glucose and other biomarker measurements, as well as self-reported information on medical treatments, disease history, and lifestyle factors. Due to funding constraints, the fourth IPT was conducted solely via a phone interview. Additionally, we established linkages to the Swedish National Patient Registry (NPR) to access diagnoses and mortality data from the Swedish Causes of Death Register (CDR), with follow-up until December 31, 2016.

### Exposures—biomarkers of epigenetic aging

Leukocyte DNA was obtained from IPT3, IPT5, IPT6, IPT8, IPT9, and IPT10. The methylation levels were measured using either the Illumina EPIC or 450k methylation arrays, following the manufacturer’s protocols. The data was pre-processed as described in a previous study [21]. The beta values were then submitted to an online methylation age calculator to generate various epigenetic estimators. These included the PAI-1 levels (DNAm PAI-1), the Hannum epigenetic clock, the Horvath1 pan-tissue clock, the PhenoAge clock, the GrimAge clock, the Horvath2 skin & blood clock, and the epigenetic measure of telomere length (DNAmTL). Additionally, DunedinPACE, an epigenetic measure of age acceleration, was generated directly using the available codes provided by its creators [22]. The PC-versions of the epigenetic clocks were generated using available codes and preferably used due to their superior test-retest reliability and longitudinal predictability. For a more detailed explanation of what these biomarkers represent, please refer to Supplementary Table 1.

### Outcome—diabetes risk

Diabetes served as the primary outcome. However, due to limitations in the international classification of diseases (ICD) codes before 1997, distinguishing between T1D and T2D was not feasible. To address this challenge, we approximated the incidence of T1D relative to T2D using data from the Swedish county of Kronoberg [2]. According to this approximation, 94% of cases in the age range of 40 to 100 years were most likely T2D cases. Our registry linkages indicated that none of the individuals had a recorded diabetes diagnosis prior to the age of 40.

Diabetes was defined as fulfilling one or more of the following criteria: (1) having an ICD-9 diagnosis in SATSA, NPR, or CDR of 250 1968–1996; (2) having an ICD-10 diagnosis in SATSA, NPR, or CDR of E10–E14 from 1997. In CDR, diabetes as a main or secondary cause of death was considered. Additionally, we applied the American Diabetes Association (ADA) criteria, which defines diabetes as blood glucose levels exceeding 7 mmol/L after an 8-h fast or 11.1 mmol/L irrespective of fasting status [5]. At each available datapoint, the individuals were classified as having or not having diabetes using ICD codes from registers, self-reported diabetes status, antidiabetic drugs (ATC codes: A10), or ADA definition using blood glucose levels. Individuals with no reported diabetes status were assumed to have no diabetes.

### Covariates

Several covariates were considered, including chronological age (CA), sex, education level, smoking status, blood glucose, and body mass index (BMI). The physical examination at the IPTs was used to evaluate BMI, which was calculated as the weight in kilograms divided by the square of the height in meters. The remaining covariates were assessed using questionnaires administered during IPT 1–10.

The study participants were classified into two categories based on their smoking status: smokers, which included both current and ex-smokers, and non-smokers. Education level was also categorized into four levels: level I for those who completed elementary school, level II for those who completed ordinary level, vocational school, or adult education, level III for those who completed secondary school or advanced level, and level IV for those who attended university or achieved a higher educational level.

There were some missing data points among the participants. Eighteen individuals did not have education data from any IPT, 45 individuals did not have recorded smoking status, another 45 lacked BMI values, and six individuals did not have blood glucose values recorded from the IPT at which their first methylation values were gathered. For these individuals, the values from the nearest IPT were used to fill in the missing data.

### Statistical analysis

We defined the baseline as the time of the first methylation measurement for everyone. Individuals diagnosed with diabetes before this baseline were categorized as having “prevalent diabetes,” while those diagnosed after were classified as “incident diabetes.” By combining these two groups, we created a third group categorized as having “diabetes.” We conducted all statistical analyses with a significance level set at *p*-value = 0.05. To characterize the study variables, we evaluated the differences in frequencies in the diabetes group, the incident diabetes group, and those without diabetes. This evaluation was carried out using chi-squared tests, Fisher’s exact tests, and paired *t*-tests.

#### Longitudinal trajectories

For each biomarker, we plotted the longitudinal trajectories of individuals who had at least one methylation measurement. These plots, encompassing a total of 1403 methylation measurements, were color-coded based on the diabetes status (at end of follow-up) of the individuals. We used locally estimated scatterplot smoothing (LOESS) to generate smoothened average (SA) curves, complete with corresponding 95% confidence bands.

#### Time to diabetes

We generated scatter plots to evaluate the relationship between the biomarkers and the time from baseline to diabetes diagnosis among the 73 individuals with incident diabetes. The biomarker data underwent Z-transformation. We used a linear regression to compute Pearson coefficients. Biomarkers such as PCPhenoAge, PCGrimAge, PCDNAmTL, PCHannum, PCHorvath1, and PCHorvath2 are expressed in biological years (refer to Supplementary Table 1), and hence, exhibit a high correlation with CA. To mitigate this correlation, we utilized their residuals, calculated as outlined previously [11]. DunedinPACE, expressed in biological year per chronological year, and DNAm PAI-1, expressed in picograms per milliliter (refer to Supplementary Table 1), do not correlate with CA to the same extent as biological years. Consequently, we did not use residuals of DunedinPACE and DNAm PAI-1 in the scatter plots.

#### Cox proportional hazards models

We employed two Cox proportional hazards (PH) models, model 1 and model 2, to discern associations between the biomarkers and diabetes mellitus (DM). Model 1, minimally adjusted for covariates, included CA, sex, and a biomarker as exposure. Model 2, fully adjusted for covariates, extended model 1 by incorporating BMI, smoking status, education level, and blood glucose level at baseline.

$$\textrm{Model}\ 1:\textrm{DM}\sim \textrm{Biomarker}\ \textrm{of}\ \textrm{aging}+\textrm{CA}+\textrm{Sex}$$

$$\textrm{Model}\ 2:\textrm{DM}\sim \textrm{Model}\ 1\ \textrm{covariates}+\textrm{BMI}+\textrm{Smoking}\ \textrm{status}+\textrm{Education}\ \textrm{level}+\textrm{Blood}\ \textrm{glucose}$$

The underlying timescale was years from baseline, and the analysis was confined to individuals with incident diabetes. We standardized the biomarkers using *Z*-transformation to render the effect sizes more comparable. The follow-up period concluded on December 31st, 2016, marking the end of the register linkage. Individuals without a diabetes diagnosis by this point were assumed to be diabetes-free and were censored at the end of the follow-up.

Each aging biomarker was fitted individually in the models. We conducted Schoenfeld tests on both model 1 and model 2 to verify the proportional hazards assumption. To evaluate the models’ performance, we calculated the mean concordance index (C-index) for both models. The C-index, a measure of a survival model’s predictive accuracy, ranges from 0 to 1. A value of 0.5 suggests the model is no better than random guessing, while a value of 1 signifies perfect prediction. In the context of Cox PH-models, the C-index is computed by comparing the predicted and actual survival times of subject pairs.

We used robust variance estimators to adjust the standard errors of the regression coefficients for the correlation between twins in the analysis. The effect sizes of the biomarkers were reported using hazard ratios (HR) and 95% confidence intervals. Point estimates were measured using estimated HRs, expressing the diabetes risk associated with a one SD increase in the biomarker.

### Sensitivity analyses

We conducted sensitivity analyses for sex and CA at baseline. For the sex-based analysis, a sub-group analysis was performed for men and women separately. For the age-based analysis, we divided the study population into two groups based on CA and fitted model 2 separately for each group. We used a cutoff point of 69 years, the median CA at baseline for the 487 individuals without prevalent diabetes. To enhance the analysis’s power, we included the same individuals in both the under-69 and over-69 groups if they had methylation measurements taken both before and after turning 69.

We also performed a sensitivity analysis on fasting status by incorporating it as a covariate in model 2, creating model 3.

$$\textrm{Model}\;3:\textrm{DM}\sim \textrm{Model}\;2\;\textrm{covariates}+\textrm{Fasting}\kern0.17em \textrm{status}\kern0.24em$$

Fifteen individuals had inconsistent missing data, with blood glucose values missing where fasting values were present, and vice versa. Specifically, twelve individuals had a blood glucose value but no fasting value, and three had a fasting value but no blood glucose value. An additional three individuals lacked both blood glucose and fasting values. Given that fasting physiologically lowers blood glucose, we used blood glucose and fasting values from the same time points to fill in the missing values for these 18 individuals in model 3. Consequently, only blood glucose and fasting values from the same time points were used in model 3.

### Sample exclusions

**Fig. 1:**
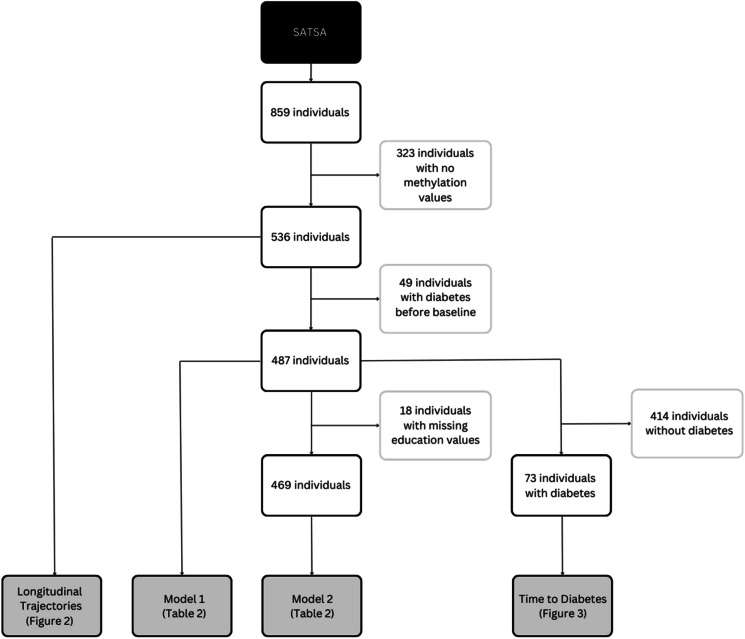
Study participant flow chart. The Swedish Adoption/Twin Study of Aging (SATSA) assessed a total of 859 individuals using IPTs. Of these, 323 individuals lacking methylation values were excluded, leaving 536 individuals. These individuals were utilized in the plots to illustrate the longitudinal biomarker trajectories. Subsequently, 49 individuals diagnosed with diabetes prior to the baseline were excluded, resulting in 487 individuals. These individuals were incorporated into the first Cox model, referred to as model 1. Model 2, which included education level as a covariate, excluded an additional 18 individuals due to missing education values. This left 469 individuals for inclusion in model 2. Finally, from the 487 individuals diagnosed with diabetes post-baseline, 414 who never developed diabetes were excluded. The remaining 73 individuals with diabetes were utilized in the plots to illustrate time to diabetes

Figure 1 presents a flow chart of the study participants. Initially, we had SATSA data from 859 individuals who participated in at least one IPT. After applying several exclusion criteria, the number of participants was reduced. This resulted in 536 individuals being included in the analysis of longitudinal trajectories, 487 in model 1, 469 in model 2, and 73 in the time-to-diabetes scatterplots.

### Ethical approval and consent to participate

The study was approved by the Swedish Ethical Review Authority with Dnr 2022-06634.

## Results

### Study characteristics

Table 1 outlines the characteristics of the 536 participants, each with at least one methylation measurement. Among these, women constituted 59% (314 individuals). On average, each participant had 2.6 methylation measurements.

**Table 1:** Study characteristics of the participants in SATSA

	Individuals without diabetes	Individuals with diabetes (incident or prevalent)	*P*-value^1^	Individuals with incident diabetes	*P*-value^1^
No. of individuals	414	122		73	
No. of methylation measurements	1091	312		213	
No. of methylation measurements per person, mean (SD)	2.6 (1.3)	2.6 (1.4)	0.58	2.9 (1.4)	0.11
Sex, *N* (%)			0.14		0.09
Women	250 (60.4)	64 (52.5)		36 (49.3)	
Men	164 (39.6)	58 (47.5)		37 (50.7)	
BMI, kg/m^2^, mean (SD)	25.8 (8.5)	27.9 (4.4)	*P* < 0.01	28.2 (4.3)	*P* < 0.01
Educational level, *N* (%)			0.76		0.18
I	219 (52.9)	66 (54.1)		34 (46.6)	
II	120 (29)	36 (29.5)		27 (37)	
III	31 (7.5)	6 (4.9)		2 (2.7)	
IV	31 (7.5)	8 (6.6)		5 (6.8)	
Missing	13 (3.1)	6 (4.9)		5 (6.8)	
Smoking status, *N* (%)			0.13		0.33
Smoker	81 (19.6)	22 (18)		12 (16.4)	
Not smoking	298 (67.2)	82 (67.2)		51 (69.9)	
Missing	35 (8.5)	18 (14.8)		10 (13.7)	
Blood glucose, mmol/L, mean (SD)	4.4 (1.1)	5.9 (2.5)	*P* < 0.01	4.8 (1.1)	0.02
Fasting before blood draw, *n* (%)	295 (71.3)	77 (63.1)	*P* < 0.01	48 (65.8)	0.02
No. of missing fasting value	14 (3.4)	8 (6.6)		1 (1.4)	
Age at diabetes onset, years, mean (SD)		73.7 (10.0)		76.8 (10.1)	
Age at baseline, years, mean (SD)	68 (9.5)	69 (9.4)	0.29	66.3 (9.4)	0.16
Age at end of follow-up, years, mean (SD)	82.7 (8.1)	73.7 (10)	*P* < 0.01	76.8 (10.1)	*P* < 0.01
*N* deaths before 2016-12-31 (%)	220 (53.1)	76 (62.3)	0.14	35 (47.9)	0.09
Mean follow-up time, years (SD)	14.8 (6.7)	4.7 (8.5)	*P* < 0.01	10.5 (5.1)	*P* < 0.01

*BMI*, body mass index; *SD*, standard deviation. The table presents the sample characteristics, which were determined prior to the use of nearest available values to fill in missing data. The term “Individuals without diabetes” refers to those who never developed diabetes either before or after the baseline, which is defined as the time of their first available methylation value. “Individuals with diabetes” encompasses those with pre-baseline or post-baseline diabetes, while “Individuals with incident diabetes” specifically refers to those who developed diabetes post-baseline. Education level was categorized into four levels: level I corresponds to “Elementary school,” level II to “O-level or vocational school or folk school,” level III to “gymnasium (A-level),” and level IV to “university or higher.” *P*-values were calculated using paired *t*-tests and Fisher’s exact tests, as appropriate. NA values were included in the Fisher’s exact tests

^1^*P*-values indicate the group differences between the group with diabetes and the group without diabetes

In the study population, individuals with diabetes, either prevalent or incident, made up 23%, with 15% being incident cases. Notably, no significant differences were observed in education level (*P* = 0.76) or smoking status (*P* = 0.13) between the group with diabetes and the group without diabetes. As expected, individuals with diabetes had higher mean blood glucose values than their diabetes-free counterparts (*P* < 0.01). A larger proportion of individuals without diabetes fasted prior to their IPTs compared to those with diabetes (*P* < 0.01).

The mean age at diabetes onset was 73.7 (SD = 10.0) years for individuals with diabetes and 76.8 (SD = 10.1) years for those with incident diabetes. No significant difference was found in the baseline age between individuals with diabetes and individuals without diabetes (*P* = 0.29). However, the mean age at the end of the follow-up was lower among individuals with diabetes (*P* < 0.01). Individuals without diabetes had a longer follow-up time of 14.8 years, compared to 10.5 years for those with incident diabetes.

### Longitudinal trajectories

In our study, we identified distinct groups when comparing individuals with diabetes (prevalent or incident) with those that did not develop diabetes during follow-up. Notably, around the age of 60, the SA curves for all biomarkers, except for PCDNAmTL, were elevated in the group with diabetes. PCDNAmTL, a biomarker that gauges telomere length and is therefore inversely associated with age, exhibited an opposite trend. Specifically, the SA curve for the group with diabetes was lower when individuals were approximately 60 years old.

Between the ages of 60 and 70, we observed significant differences for DunedinPACE and DNAm PAI-1, with no overlap in confidence intervals. The SA curves for DunedinPACE, PCDNAmTL, PCHannum, PCHorvath1, and PCHorvath2 started to show convergence around the age of 65. This trend continued with the SA curves of PCGrimAge converging at 75 years, while those of PCPhenoAge and DNAm PAI-1 did not show convergence until the individuals reached 80 years of age.

**Fig. 2:**
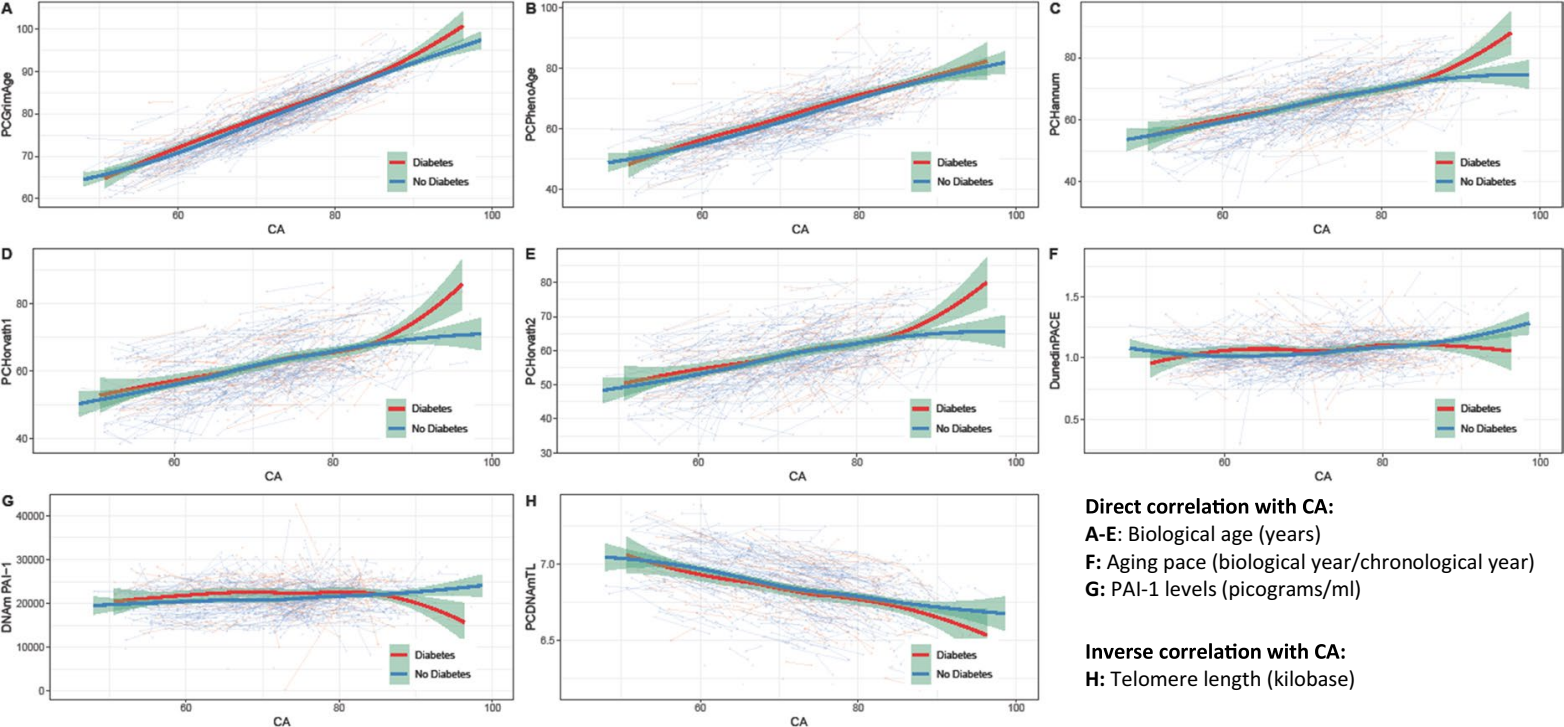
Longitudinal biomarker trajectories. A total of 859 individuals from the Swedish Twin Study of Aging (SATSA) participated in at least one in-person testing occasion. From these, 323 individuals lacking methylation values were excluded, resulting in 536 participants for the study. Each plot incorporates 1403 methylation measurements. It is important to note that the units on the *y*-axis differ for each biomarker (refer to Supplementary Table 1 for details). The trajectories are color-coded: red for individuals who developed diabetes and blue for those who did not. Smoothened average (SA) curves were constructed using the locally estimated scatterplot smoothening (LOESS) method, with 95% confidence intervals marked in green. The findings indicate that around the age of 60, SA values for diabetic individuals were higher for all biomarkers, except for PCDNAmTL which measures telomere length and has an inverse relation with age. The differences were statistically significant for the biomarkers DunedinPACE and DNAm PAI-1 during the age range of 60–70 years. Post the age of 85, all the curves diverged, likely due to fewer measurements

However, after the age of 85, a divergence in all curves was noted, likely due to fewer measurements. Of particular note, PCGrimAge demonstrated poor accuracy in predicting CA, as it overestimated the biological age for most individuals (Figure 2).

### Time to diabetes

**Fig. 3:**
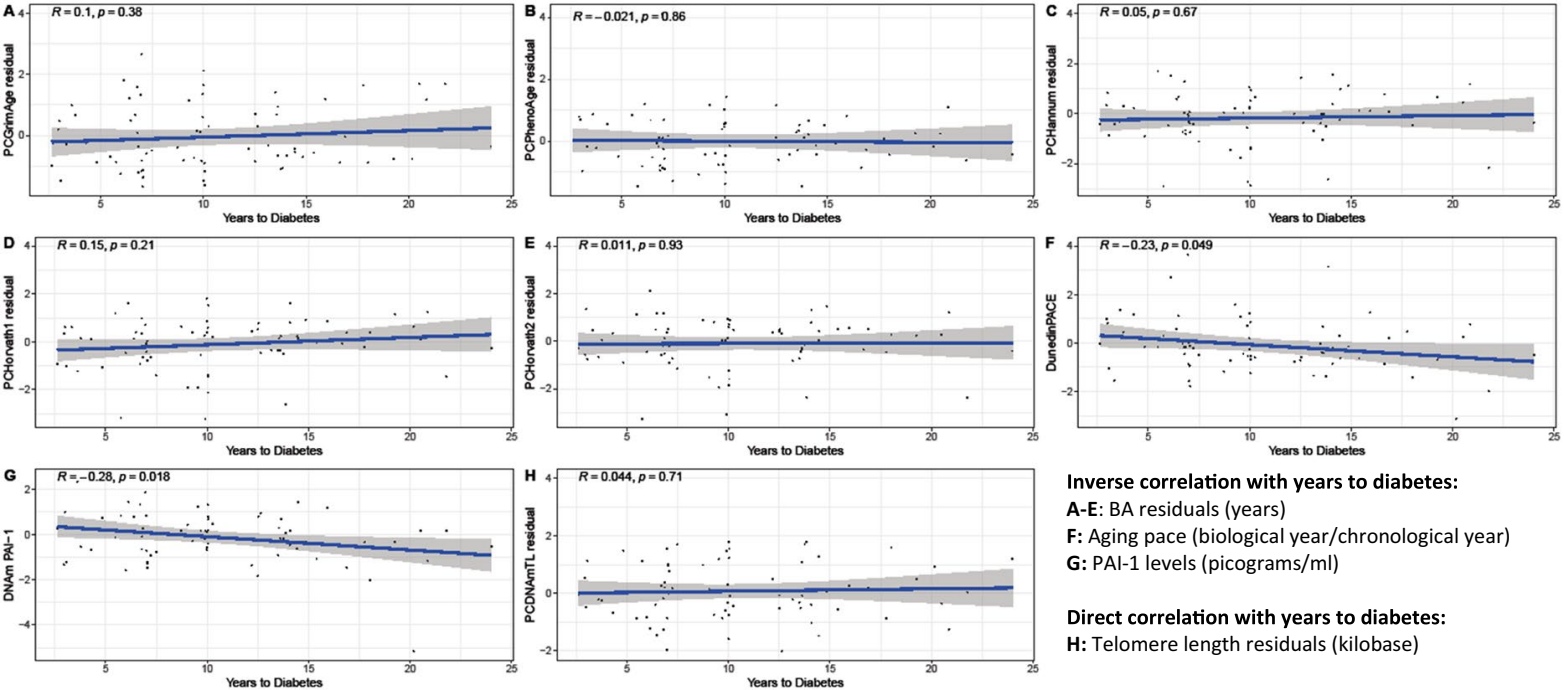
Correlation of biomarkers with time to diabetes diagnosis. This figure presents the results of a linear regression analysis examining the association between various biomarkers and the time to diabetes diagnosis. The analysis was conducted on a subset of 73 individuals who were diagnosed with diabetes after the baseline measurement. The first available methylation value was used to generate the biomarker values for each individual. Biomarkers such as PCPhenoAge, PCGrimAge, PC DNAmTL, PCHannum, PCHorvath1, and PCHorvath2 are expressed in biological years (refer to Supplementary Table 1), which highly correlate with chronological age. To mitigate this correlation, residuals of these biomarkers were used in the analysis. On the other hand, DunedinPACE (expressed in biological year per chronological year) and DNAm PAI-1 (expressed in picograms per milliliter) do not correlate with chronological age as strongly as the aforementioned biomarkers. Consequently, no residuals were used for these two biomarkers in the analysis. Pearson coefficients, denoted as *R*, were used to evaluate the models. Prior to regression, the biomarkers were *Z*-transformed to enhance comparability. The analysis revealed a negative correlation between time to diabetes diagnosis and both DunedinPACE and DNAm PAI-1

Figure 3 depicts the correlations between the years to a diabetes diagnosis and the biomarkers adjusted for CA. This analysis was confined to the 73 individuals who were diagnosed with diabetes during the study. Notably, DunedinPACE and DNAm PAI-1 exhibited significant negative correlations with the years to diabetes diagnosis, with Pearson coefficients of −0.23 and −0.28 respectively. However, no significant linear correlation was observed between the remaining biomarkers and the time to diabetes diagnosis.

### Cox proportional hazards models

In our analysis, both model 1 and model 2 (refer to Table 2) failed to establish any significant associations between biomarkers of epigenetic aging and diabetes. The confidence intervals were wide, indicating limited power. The mean C-index was 0.59 (SD=0.003) for model 1 and 0.66 (SD=0.003) for model 2. The elevated C-index for model 2 indicates an improved model fit, attributable to the inclusion of additional covariates.

**Table 2:** Results of Cox-PH regression models

Biomarker of aging	Model 1: HR (CI)	Model 2: HR (CI)
DNAm PAI-1	1.09 (0.84, 1.41)	1.00 (0.76, 1.30)
PCDNAmTL	0.91 (0.69, 1.20)	0.97 (0.71, 1.31)
DunedinPACE	1.11 (0.88, 1.40)	1.07 (0.82, 1.40)
PCHorvath1	0.98 (0.76, 1.27)	0.95 (0.72, 1.26)
PCHorvath2	1.04 (0.81, 1.34)	0.98 (0.75, 1.29)
PCHannum	0.96 (0.73, 1.26)	0.90 (0.67, 1.21)
PCPhenoAge	1.02 (0.68, 1.55)	1.04 (0.67, 1.62)
PCGrimAge	1.06 (0.58, 1.94)	0.96 (0.47, 1.99)
Mean C-index	0.59 (SD 0.003)	0.66 (SD 0.003)

*HR*, hazard ratio; *CI*, 95% confidence interval; *C-index*, concordance index; *SD*, standard deviation. The table presents the outcomes of the Cox proportional hazards models. In model 1, the first diabetes model, adjustments were made solely for chronological age and sex. Conversely, model 2, the second diabetes model, accounted for additional factors such as BMI, smoking status, education level, and blood glucose levels

### Sensitivity analysis

Schoenfeldt tests were employed to verify the assumption of proportional hazards, and the results confirmed that the hazards were indeed proportional over time (refer to Supplementary Table 2).

When the models were separately fitted for men and women, distinct patterns emerged. Women generally exhibited higher estimates, while men had lower estimates (see Supplementary Table 3). In model 2, all diabetes-risk associations for women, excluding DNAm PAI-1, were stronger. Notably, PCPhenoAge and PCDNAmTL demonstrated statistically significant diabetes-risk associations, with HRs (95% CI) of 2.86 (1.41, 5.77) and 0.61 (0.38, 0.97), respectively. Conversely, for men in model 2, all diabetes-risk associations, except for DNAm PAI-1, were weaker. PCHorvath1, PCHorvath2, and PCHannum showed significantly inverse diabetes-risk associations, with HRs of 0.62 (0.41, 0.95), 0.65 (0.43, 0.97), and 0.60 (0.39, 0.93), respectively.

No significant associations were observed for individuals either under or over 69 years of age (Supplementary Table 4). A sensitivity analysis for fasting status revealed that its inclusion as a covariate had a non-significant impact on the estimates, with associations remaining insignificant when fasting was accounted for (Supplementary Table 5).

## Discussion

This study delved into the investigation of eight epigenetic biomarkers of aging and their correlation with diabetes. It was observed that the SA curves of DunedinPACE and DNAm PAI-1 for individuals who had or developed diabetes were higher than those who did not, specifically in the age group of 60–70 years. Interestingly, only DunedinPACE and DNAm PAI-1 were higher closer to diabetes onset in linear regression. However, none of the biomarkers demonstrated a statistically significant association with diabetes risk in the Cox PH models. A notable difference was observed when the models were run separately for men and women, with women generally showing stronger associations reaching statistical significance and men showing weaker ones.

Our findings align with those of Fraszczyk et al., who explored the relationship of biomarkers GrimAge, Horvath, Hannum, and PhenoAge with T2D, 10 years prior to T2D onset [18]. They found that the longitudinal trajectories of individuals with T2D were higher than those of controls up to 10 years before T2D onset, albeit the difference was not statistically significant. Furthermore, they found no associations between biomarker residuals and time to diabetes for GrimAge, Horvath, Hannum, and PhenoAge.

Our study diverges from that of Fraszczyk et al. in several ways. We employed different analytical methods, incorporated additional epigenetic biomarkers, used PC-versions of the biomarkers, had a longer follow-up period, and our sample had a mean age that was 8 years higher. We also separately investigated the associations of the biomarkers in men and women, which revealed stronger associations in women and weaker ones in men. This is consistent with a previous study in the same cohort, which found stronger mortality associations in women for most of the biomarkers used [11]. One plausible explanation could be the differential aging process in men and women, which is reflected in their epigenetic landscape [23]. However, due to the small sample sizes for each sex and large confidence intervals, it is challenging to conclusively determine any differences between the sexes.

The intricate links between aging, epigenetics, and T2D are manifold. Previous studies have shown that epigenetic changes are associated with T2D [17, 18]. DNA methylation, which influences gene expression, may play a role in the pathogenesis of T2D. However, it remains unclear whether changes in the DNA methylome are a cause or a consequence of T2D. It has been proposed that senescent cells, which accumulate in many tissues with age, may play a role in the pathogenesis of T2D [9]. These senescent cells secrete senescence-associated secretory phenotypes (SASPs), such as activin A, IL-6 and TNF-alpha, that induce insulin resistance [8, 24, 25]. This could partially explain why T2D shows a strong association with CA.

Speculation arises as to why DNAm PAI-1 and DunedinPACE demonstrated the most potent associations with diabetes. PAI-1 is mainly secreted by endothelial cells, hepatocytes, and fat cells [26]. PAI-1 inhibits fibrinolysis and is thus associated with blood clots and atherosclerosis [27]. Although the exact role of PAI-1 in T2D remains elusive, several mechanisms have been proposed [26]. For instance, PAI-1 has been shown to predict metabolic syndrome [28, 29], which is associated with T2D [30]. Additionally, PAI-1 contributes to insulin resistance, which in turn stimulates PAI-1 secretion [31, 32], suggesting a bi-directional relationship between PAI-1 and insulin resistance [33]. Experimental studies in both animals and humans have shown that glucose upregulates PAI-1 gene expression in vascular smooth muscle cells, endothelial cells, and adipose tissue [34–37]. It is well known that glucose is elevated among individuals with prediabetes [38], which may explain why we found associations between DNAm PAI-1 and diabetes. DunedinPACE, previously linked to chronic diseases such as T2D [22], is unique in that it was trained on multiple longitudinal measurements, unlike other biomarkers trained on cross-sectional measurements. This could account for DunedinPACE’s superior performance in longitudinal analyses. The biomarker levels of DunedinPACE and DNAm PAI-1 were larger when a diabetes diagnosis was imminent (Figure 3) and around the age of 60–70 (Figure 2). This coincides with the mean diabetes onset age of 73.7 years (Table 1), suggesting that individuals may have had prediabetes or undiagnosed diabetes in the years leading up to the diagnosis, resulting in a deteriorated health status and higher biological age. Thus, it seems that the epigenetic changes related to diabetes become mainly detectable within a limited time before the diabetes diagnosis, aligning with the fact that T2D is a gradual disease, with patients often developing prediabetes years before T2D is diagnosed [38].

The present study has several strengths. Firstly, the study utilized eight biomarkers in the same population, which gave us the opportunity to investigate how the biomarkers compare to each other. Secondly, longitudinal data was used to see how the biomarkers performed at different ages. Thirdly, we were able to track the diabetes status and survival status of individuals for a long period, namely 1984–2016, although the biomarkers were only available from IPT 3 (1992). This provided an opportunity to track changes in biomarkers in relation to diabetes, from midlife through late life.

Our study is subject to several limitations. The first pertains to the inability to distinguish between T1D and T2D due to the lack of distinction in ICD codes prior to 1997. We made an assumption based on the T1D to T2D ratio found in the Swedish county of Kronoberg, which was approximately 1:16 for incident cases aged 40 to 100 [2]. Given that all cases fell within this age range, we estimated that 94% of cases in our models were likely T2D. However, this approximation is constrained by the absence of primary care linkages, suggesting that some individuals might have developed diabetes earlier without our knowledge.

The second limitation of our study stems from the exclusion of key covariates such as age, BMI, smoking status, education level, and blood glucose level in the linear regression analysis conducted to ascertain the relationship between the biomarkers and the time to T2D. This exclusion presents a challenge in conclusively determining whether DNAm PAI-1 and DunedinPACE are simply superior indicators of these covariates compared to other biomarkers. This is particularly significant given the already established correlation between these factors and T2D [5, 39, 40].

The third limitation is the potential for survival bias inherent in the longitudinal design of our study. Additionally, the wide birth year range of the SATSA participants (1900–1948) introduces variability in medical advancements and living conditions experienced by individuals.

Lastly, the large confidence intervals in our study, likely due to the limited sample size and inherent limitations of epigenetic biomarkers, pose another limitation. For instance, Illumina microarrays capture CpG methylation at a cell population level, not at a single-cell level [41], potentially reducing the utility of epigenetic biomarkers as changes may occur in only a subset of cells. The wide confidence intervals could also be attributed to the fact that the epigenetic biomarkers were trained on cohorts different from the SATSA cohort.

To conclude, the causal relationship between prediabetes and the epigenetic changes detected by these biomarkers remains uncertain. Our findings suggest the potential value of developing epigenetic biomarkers specifically tailored to T2D, should we wish to model and explore the potential for predicting the disease.

### Supplementary information

ESM 1 (DOCX 28.7 kb)

## Data Availability

Methylation data are available in EMBL-EBI under accession number S-BSST1206 (https://www.ebi.ac.uk/biostudies/studies/S-BSST1206), whereas phenotypic data are available in the National Archive of Computerized Data on Aging under accession number ICPSR 3843 (https://www.icpsr.umich.edu/web/NACDA/studies/3843).
