# Supplementary material for: Associations between epigenetic aging and diabetes mellitus in a Swedish longitudinal study
Source: GeroScience. 2024 Jun 27;46(5):5003–14. doi: 10.1007/s11357-024-01252-7 (PMC11335983; doi:10.1007/s11357-024-01252-7)
Supplement: Supplementary file 1 — (DOCX 28.7 kb) [file 11357_2024_1252_MOESM1_ESM.docx]

**Supplementary Files**

Supplementary Table 1. Characteristics of the Biomarkers

| **Biomarker of aging** | **Phenotypes used for training** | **Measurement method** | **Component elements** | **Method** | **Unit of measure** | **Principal component (PC) version available** | **Source** |
| --- | --- | --- | --- | --- | --- | --- | --- |
| DNAm PAI-1 | PAI-1 levels | Illumina EPIC or 450k methylation arrays | 211 CpGs | Elastic net to regress PAI-1 levels on CpGs. | PAI-1 levels (pg/ml) | No | (1) |
| DNAmTL | Telomere length |  | 140 CpGs | Elastic net to regress telomere length on CpGs | Telomere length (kilobase) | Yes | (2) |
| DunedinPACE | BMI, Waist-hip ratio, Glycated hemoglobin, Leptin, Blood pressure (mean arterial pressure), VO2Max, FEV1/FVC, FEV1, Total cholesterol, Triglycerides, HDL, Lipoprotein(a), Apolipoprotein B100/A1 ratio, eGFR, BUN, hs-CRP, White blood cell count, AL, and tooth decay |  | 173 CpGs | Step 1: Linear mixed-effects modelling to estimate rate of change for each of the 19 biomarkers.  Step 2: Elastic-net-regression to predict the Pace of Aging from the rate of change of the biomarkers. | Pace of aging (biological years per chronological year) | No | (3) |
| Horvath1 | CA |  | 353 CpGs | Elastic net to regress CA on CpGs | Biological age (biological age in years) | Yes | (4) |
| Horvath2 | CA |  | 391 CpGs | Elastic net to regress CA on CpGs |  | Yes | (5) |
| Hannum | CA |  | 71 CpGs | Elastic net to regress CA on CpGs |  | Yes | (6) |
| PhenoAge | CA, albumin, creatinine, glucose, CRP, lymphocyte percentage, mean cell volume, red blood cell distribution width, alkaline phosphatase, white blood cell count |  | 513 CpGs | Step 1: Penalized proportional hazards to regress time-to-death on clinical markers and CA.  Step 2: Elastic net to regress on CpGs |  | Yes | (7) |
| GrimAge | CA, Adrenomedullin, beta-2 microglobulin, growth differentiation factor 15, Cystatin C, leptin, PAI-1, and tissue inhibitor metalloproteinase 1 and smoking pack-years |  | Smoking pack years and 1030 plasma protein associated CpGs | Step 1: Elastic net to regress biomarkers on CpGs.  Step 2: Elastic net Cox to regress time-to-death on DNAm-based biomarkers and CA. |  | Yes | (1) |

**BMI, body mass index; VO2Max, cardiorespiratory fitness; FEV1/FVC, Forced vital capacity ratio; FEV1, Forced expiratory volume in one second; HDL, High density lipoprotein; eGFR, estimated Glomerular Filtration Rate; BUN, Blood Urea Nitrogen; hs-CRP High Sensitivity C-reactive Protein; AL, mean periodontal attachment loss; CA, chronological age; C-reactive protein levels, CRP; PAI-1, plasminogen activation inhibitor 1; BA, biological age.**

1. Lu AT, Quach A, Wilson JG, Reiner AP, Aviv A, Raj K, et al. DNA methylation GrimAge strongly predicts lifespan and healthspan. Aging (Albany NY). 2019 Jan 21;11(2):303–27.

2. Lu AT, Seeboth A, Tsai PC, Sun D, Quach A, Reiner AP, et al. DNA methylation-based estimator of telomere length. Aging (Albany NY). 2019 Aug 18;11(16):5895–923.

3. Belsky DW, Caspi A, Corcoran DL, Sugden K, Poulton R, Arseneault L, et al. DunedinPACE, a DNA methylation biomarker of the pace of aging. Deelen J, Tyler JK, Suderman M, Deelen J, editors. eLife. 2022 Jan 14;11:e73420.

4. Horvath S. DNA methylation age of human tissues and cell types. Genome Biol. 2013;14(10):R115.

5. Horvath S, Oshima J, Martin GM, Lu AT, Quach A, Cohen H, et al. Epigenetic clock for skin and blood cells applied to Hutchinson Gilford Progeria Syndrome and ex vivo studies. Aging (Albany NY). 2018 Jul 26;10(7):1758–75.

6. Hannum G, Guinney J, Zhao L, Zhang L, Hughes G, Sadda S, et al. Genome-wide methylation profiles reveal quantitative views of human aging rates. Mol Cell. 2013 Jan 24;49(2):359–67.

7. Levine ME, Lu AT, Quach A, Chen BH, Assimes TL, Bandinelli S, et al. An epigenetic biomarker of aging for lifespan and healthspan. Aging (Albany NY). 2018 Apr 17;10(4):573–91.

Supplementary Table 2. Results of Schoenfeld Test

| **Biomarker Model** | **Model 1: P-value** | **Model 2: P-value** |
| --- | --- | --- |
| DNAm PAI-1 | 0.13 | 0.14 |
| PCDNAmTL | 0.87 | 0.12 |
| DunedinPACE | 0.23 | 0.06 |
| PCHorvath1 | 0.61 | 0.15 |
| PCHorvath2 | 0.84 | 0.15 |
| PCHannum | 0.79 | 0.15 |
| PCPhenoAge | 0.87 | 0.14 |
| PCGrimAge | 0.52 | 0.15 |

**HR, hazard ratio. A Schoenfeld test was conducted on each model. The table shows the resulting P-value for each biomarker model.**

Supplementary Table 3. Sensitivity Analysis for Sex

| **Biomarker of aging** | **Women (N = 256)** | **Men (N = 201)** |
| --- | --- | --- |
| DNAm PAI-1 | 0.89 (0.64, 1.24) | 1.06 (0.71, 1.57) |
| PCDNAmTL | 0.61 (0.38, 0.97) | 1.30 (0.86, 1.97) |
| DunedinPACE | 1.37 (0.95, 1.99) | 0.84 (0.57, 1.26) |
| PCHorvath1 | 1.47 (0.99, 2.19) | 0.62 (0.41, 0.95) |
| PCHorvath2 | 1.46 (0.97, 2.20) | 0.65 (0.43, 0.97) |
| PCHannum | 1.41 (0.88, 2.23) | 0.60 (0.39, 0.93) |
| PCPhenoAge | 2.86 (1.41, 5.77) | 0.59 (0.31, 1.10) |
| PCGrimAge | 2.63 (0.91, 7.58) | 0.47 (0.17, 1.24) |

**HR, Hazard Ratio; CI, 95% Confidence Interval. The table shows the results of Model 2 when it is run on men and women separately.**

Supplementary Table 4. Sensitivity Analysis for Chronological Age

| **Biomarker of aging** | **Under 69 years (N = 256)** | **Over 69 years (N = 369)** |
| --- | --- | --- |
|  |  |  |
| DNAm PAI-1 | 1.15 (0.80, 1.67) | 0.93 (0.68, 1.28) |
| PCDNAmTL | 1.00 (0.65, 1.53) | 0.88 (0.62, 1.26) |
| DunedinPACE | 1.13 (0.80, 1.58) | 1.16 (0.82, 1.65) |
| PCHorvath1 | 1.00 (0.69, 1.44) | 0.98 (0.68, 1.41) |
| PCHorvath2 | 1.01 (0.70, 1.47) | 1.03 (0.74, 1.44) |
| PCHannum | 0.89 (0.60, 1.34) | 0.96 (0.67, 1.39) |
| PCPhenoAge | 1.16 (0.64, 2.09) | 1.10 (0.63, 1.91) |
| PCGrimAge | 1.15 (0.47, 2.82) | 1.24 (0.48, 3.22) |
|  |  |  |

**HR, Hazard ratio; 95% confidence interval. The table shows the results of Model 2 when it is run on methylation measurements taken when the individuals were under 69 and over 69 respectively.**

Supplementary Table 5. Sensitivity Analysis for Fasting Status

| **Biomarker of aging** | **Without Fasting** | **With Fasting** |
| --- | --- | --- |
|  |  |  |
| DNAm PAI-1 | 1.00 (0.77, 1.30) | 1.01 (0.77, 1.31) |
| PCDNAmTL | 0.96 (0.71, 1.31) | 0.97 (0.71, 1.31) |
| DunedinPACE | 1.07 (0.82, 1.38) | 1.06 (0.82, 1.37) |
| PCHorvath1 | 0.95 (0.72, 1.26) | 0.96 (0.73, 1.27) |
| PCHorvath2 | 0.98 (0.75, 1.29) | 0.99 (0.75, 1.30) |
| PCHannum | 0.90 (0.67, 1.21) | 0.90 (0.67, 1.21) |
| PCPhenoAge | 1.04 (0.67, 1.62) | 1.04 (0.67, 1.61) |
| PCGrimAge | 0.97 (0.47, 2.00) | 0.94 (0.45, 1.94) |

**HR, Hazard ratio; CI, 95% confidence interval. The table shows the results of a sensitivity analysis done for fasting using Cox proportional hazards modelling. The model was referred to as Model 3.**
